# Supplementary material for: The Roles of Mating, Age, and Diet in Starvation Resistance in Bactrocera oleae (Olive Fruit Fly)
Source: Insects. 2023 Oct 29;14(11):841. doi: 10.3390/insects14110841 (PMC10672083; doi:10.3390/insects14110841)
Supplement: Supplementary file 1 [file insects-14-00841-s001.zip › insects-2628724-supplementary.pdf]

Supplementary Table 1 (S1). Tukey (HSD) results for males. Mean values followed by different numbers are statistically significant different, at significance level *a*=0.05 (*p*≤0.05), according to the results of the Tukey's multiple comparisons procedure.

Mating Status,		Subset							
Diet, Age class*	N	1	2	3	4	5	6	7	8
M. F. [31 – 40]	10	25.060							
M. F. [21 – 30]	10	27.160	27.160						
M. F. [41 – 50]	10	30.000	30.000						
M. R. [31 – 40]	10	33.820	33.820	33.820					
V. F. [41 – 50]	10	39.610	39.610	39.610	39.610				
M. F. [11 – 20]	10	41.000	41.000	41.000	41.000	41.000			
M. R. [41 – 50]	10	41.400	41.400	41.400	41.400	41.400	41.400		
V. F. [31 – 40]	10		48.450	48.450	48.450	48.450	48.450	48.450	
M. R. [21 – 30]	10			53.260	53.260	53.260	53.260	53.260	53.260
V. R. [21 – 30]	10			54.380	54.380	54.380	54.380	54.380	54.380
V. R. [41 – 50]	10				57.530	57.530	57.530	57.530	57.530
V. F. [21 – 30]	10					63.290	63.290	63.290	63.290
V. R. [31 – 40]	10						64.000	64.000	64.000
M. R. [11 – 20]	10							65.820	65.820
V. F. [11 – 20]	10								72.030
V. R. [11 – 20]	10								72.240
Sig.		.460	.089	.120	.300	.058	.051	.352	.214

*For mating status: V: virgin, M: mated, for diet: F: full, R: restricted. Means for groups in homogeneous subsets are displayed.

Supplementary Table 2 (S2). Tukey (HSD) results for females. Mean values followed by different numbers are statistically significant different, at significance level *a*=0.05 (*p*≤0.05), according to the results of the Tukey's multiple comparisons procedure.

Mating Status, Diet,		Subset								
Age Classes*	N	1	2	3	4	5	6	7	8	9
M. F. [41 – 50]	10	32.000								
M. R. [41 – 50]	10	33.383								
M. F. [31 – 40]	10	33.590	33.590							
M. R. [31 – 40]	10	42.280	42.280	42.280						
M. F. [21 – 30]	10	43.410	43.410	43.410						
V. F. [41 – 50]	10	46.510	46.510	46.510	46.510					
V. F. [31 – 40]	10	53.300	53.300	53.300	53.300	53.300				
M. F. [11 – 20]	10		57.400	57.400	57.400	57.400	57.400			
V. F. [21 – 30]	10			63.670	63.670	63.670	63.670	63.670		
V. R. [31 – 40]	10				68.980	68.980	68.980	68.980	68.980	
M. R. [21 – 30]	10					70.860	70.860	70.860	70.860	
V. F. [11 – 20]	10					74.350	74.350	74.350	74.350	
M. R. [11 – 20]	10						78.860	78.860	78.860	
V. R. [21 – 30]	10							86.020	86.020	86.020
V. R. [41 – 50]	10								88.300	88.300
V. R. [11 – 20]	10									107.790
Sig.		.142	.053	.138	.092	.156	.134	.096	.272	.120

*For mating status: V: virgin, M: mated, for diet: F: full, R: restricted. Means for groups in homogeneous subsets are displayed.

Supplementary Table 3 (S3). Additional descriptive statistical indices for each treatments' combination for Starvation Resistance for males.

Mating Status	Diet	Age Classes	Minimum	Median	Maximum	Mean	Std. Error of Mean	Std. Deviation	N
Virgin	Full	11-20	59.9	70.9	98.3	72.0	3.6	11.5	10
		21-30	42.7	60.9	111.2	63.3	5.7	18.1	10
		31-40	37.3	42.4	63.2	48.5	3.2	10.2	10
		41-50	34.5	38.8	48.1	39.6	1.4	4.3	10
	Restricted	11-20	57.6	75.0	82.1	72.2	2.5	7.8	10
		21-30	35.2	54.6	68.0	54.4	3.1	9.9	10
		31-40	35.9	70.8	94.0	64.0	6.7	21.2	10
		41-50	38.2	59.9	73.5	57.5	3.8	12.1	10
Mated	Full	11-20	31.7	39.3	62.0	41.0	5.5	12.3	10
		21-30	19.3	27.9	36.0	27.2	1.8	5.8	10
		31-40	16.3	23.7	38.7	25.1	2.5	8.1	10
		41-50	30.0	30.0	30.0	30.0	.0	.0	10
	Restricted	11-20	55.0	63.7	81.7	65.8	4.6	10.3	10
		21-30	31.7	53.5	76.3	53.3	4.8	15.2	10
		31-40	17.7	33.2	51.0	33.8	3.1	9.9	10
		41-50	29.0	34.0	56.7	41.4	5.8	13.0	10

Supplementary Table 4 (S4). Additional descriptive statistical indices for each treatments' combination for Starvation Resistance for females.

Mating Status	Diet	Age Classes	Minimum	Median	Maximum	Mean	Std. Error of Mean	Std. Deviation	N
Virgin	Full	11-20	66.8	71.0	89.6	74.4	2.4	7.7	10
		21-30	50.5	64.0	75.0	63.7	2.6	8.1	10
		31-40	45.3	52.0	62.6	53.3	1.7	5.4	10
		41-50	33.8	47.9	50.4	46.5	1.5	4.9	10
	Restricted	11-20	85.5	110.2	124.4	107.8	3.7	11.8	10
		21-30	58.2	89.8	122.0	86.0	6.5	20.4	10
		31-40	51.0	65.4	96.4	69.0	4.8	15.3	10
		41-50	55.8	93.5	119.7	88.3	7.1	22.4	10
Mated	Full	11-20	46.7	55.7	76.0	57.4	5.0	11.2	10
		21-30	33.3	46.7	51.3	43.4	2.2	6.9	10
		31-40	22.7	31.8	56.3	33.6	3.6	11.2	10
		41-50	24.0	32.0	40.0	32.0	4.6	8.0	10
	Restricted	11-20	58.3	83.0	103.0	78.9	7.8	17.3	10
		21-30	45.0	67.2	111.3	70.9	6.9	21.9	10
		31-40	31.7	42.2	57.0	42.3	2.7	8.4	10
		41-50	19.0	32.7	43.3	33.4	3.6	8.9	10

Supplementary Table 5 (S5). Tests of Between-Subjects Effects for males (Dependent Variable: Starvation resistance in hours)

Source	Type III Sum of Squares	df	Mean Square	F	Sig.	Partial Eta Squared	Noncent. Parameter	Observed Power[b]
Corrected Model	32349.325[a]	15	2156.622	14.825	.000	.648	222.377	1.000
Intercept	270695.610	1	270695.610	1860.829	.000	.939	1860.829	1.000
Mating Status	10312.644	1	10312.644	70.892	.000	.369	70.892	1.000
Diet	3994.445	1	3994.445	27.459	.000	.185	27.459	.999
Age Classes	7425.427	3	2475.142	17.015	.000	.297	51.044	1.000
Mating Status * Diet	932.442	1	932.442	6.410	.013	.050	6.410	.709
Mating Status * Age Classes	709.828	3	236.609	1.627	.187	.039	4.880	.418
Diet * Age Classes	139.837	3	46.612	.320	.811	.008	.961	.111
Mating Status * Diet * Age Classes	2836.905	3	945.635	6.501	.000	.139	19.502	.967
Error	17601.921	144	145.470					
Total	398417.000	160						
Corrected Total	49951.246	159						

[a] R Squared = .648 (Adjusted R Squared = .604), [b] Computed using alpha = .05.

Supplementary Table 6 (S6). Tests of Between-Subjects Effects for females (Dependent Variable: Starvation resistance in hours)

Source	Type III Sum of Squares	df	Mean Square	F	Sig.	Partial Eta Squared	Noncent. Parameter	Observed Power[b]
Corrected Model	64029.583[a]	15	4268.639	23.885	.000	.744	358.281	1.000
Intercept	457990.013	1	457990.013	2562.711	.000	.954	2562.711	1.000
Mating Status	18506.126	1	18506.126	103.552	.000	.457	103.552	1.000
Diet	14127.508	1	14127.508	79.051	.000	.391	79.051	1.000
Age Classes	18217.273	3	6072.424	33.979	.000	.453	101.936	1.000
Mating Status * Diet	1402.836	1	1402.836	7.850	.006	.060	7.850	.794
Mating Status * Age Classes	1056.702	3	352.234	1.971	.122	.046	5.913	.498
Diet * Age Classes	1208.217	3	402.739	2.254	.086	.052	6.761	.558
Mating Status * Diet * Age Classes	1919.752	3	639.917	3.581	.016	.080	10.742	.779
Error	21981.714	144	178.713					
Total	639114.400	160						
Corrected Total	86011.298	159						

[a] R Squared = .744 (Adjusted R Squared = .713), [b] Computed using alpha = .05
